# Ethanol Seeking by Long Evans Rats Is Not Always a Goal-Directed Behavior

**DOI:** 10.1371/journal.pone.0042886

**Published:** 2012-08-03

**Authors:** Regina A. Mangieri, Roberto U. Cofresí, Rueben A. Gonzales

**Affiliations:** College of Pharmacy, Division of Pharmacology and Toxicology, The University of Texas at Austin, Austin, Texas, United States of America; Radboud University, Netherlands

## Abstract

**Background:**

Two parallel and interacting processes are said to underlie animal behavior, whereby learning and performance of a behavior is at first *via* conscious and deliberate (goal-directed) processes, but after initial acquisition, the behavior can become automatic and stimulus-elicited (habitual). With respect to instrumental behaviors, animal learning studies suggest that the duration of training and the action-outcome contingency are two factors involved in the emergence of habitual seeking of “natural” reinforcers (e.g., sweet solutions, food or sucrose pellets). To rigorously test whether behaviors reinforced by abused substances such as ethanol, in particular, similarly become habitual was the primary aim of this study.

**Methodology/Principal Findings:**

Male Long Evans rats underwent extended or limited operant lever press training with 10% sucrose/10% ethanol (10S10E) reinforcement (variable interval (VI) or (VR) ratio schedule of reinforcement), or with 10% sucrose (10S) reinforcement (VI schedule only). Once training and pretesting were complete, the impact of outcome devaluation on operant behavior was evaluated after lithium chloride injections were paired with the reinforcer, or unpaired 24 hours later. After limited, but not extended instrumental training, lever pressing by groups trained under VR with 10S10E and under VI with 10S was sensitive to outcome devaluation. In contrast, responding by both the extended and limited training 10S10E VI groups was not sensitive to ethanol devaluation during the test for habitual behavior.

**Conclusions/Significance:**

Operant behavior by rats trained to self-administer an ethanol-sucrose solution showed variable sensitivity to a change in the value of ethanol, with relative insensitivity developing sooner in animals that received time-variable ethanol reinforcement during training sessions. One important implication, with respect to substance abuse in humans, is that initial learning about the relationship between instrumental actions and the opportunity to consume ethanol-containing drinks can influence the time course for the development or expression of habitual ethanol seeking behavior.

## Introduction

Many of the criteria used to diagnose substance use disorders in humans describe an impaired ability to regulate or to refrain from behaviors instrumental in obtaining or consuming the reinforcing substance, despite the individual's desire to do so [1]. This aspect of addiction is captured by its characterization as a neurobiological and behavioral pathology in which cognitive expectations and conscious desires appear to have a diminished role in guiding decision making and behaviors related to the addictive substance [2], [3]. To explain such apparently paradoxical behavior, Tiffany applied the cognitive psychology concept of automaticity to argue that drug use behaviors by addicts are like any other well-practiced behavior and can come to be under the control of automatic cognitive processes [2]. Automatic processes underlie behaviors that are stimulus-elicited, initiated without intention, and are able to be completed without conscious awareness, requiring little cognitive effort [2]. Non-automatic processes, on the other hand, are engaged when behavior is conscious and controlled, and these two types of cognitive processes have been likened to the proposed dual neural pathways, described by studies of reinforcement learning and instrumental behavior, that are responsible for habitual and goal-directed behaviors [4].

As a brief aside, ‘automatic’, or ‘habitual’, and ‘non-automatic’, or ‘goal-directed’, processes are used here with the intention that they be understood as working models to describe brain function, not necessarily as accurate accounts of tangible reality. Nonetheless, converging evidence indicates that these hypothetical processes do indeed arise from existent, functionally definable, neuroanatomical pathways that are responsible for learning, decision-making, and behavioral execution [3], [5], [6], [7]. Initially, a behavior becomes a goal-directed action when the outcome of the behavior itself stimulates further performance of the instrumental action [5], [8]. Compared to the habitual responses that develop later, goal-directed actions are more dependent upon feedback from the reinforcer to stimulate their performance, and therefore, are more affected when the value of the operant reinforcer changes [8]. Thus, habits, which are elicited independently of the reinforcer by reinforcer-associated stimuli, can be operationally distinguished on the basis of their relative insensitivity to changes in the value of their outcome [7]. To observe this differential sensitivity, behavioral testing is conducted without reinforcer feedback (i.e., under extinction conditions) after the value of instrumental outcome has been manipulated [7]. A subsequent test in which operant responses do receive response-contingent deliveries of the revalued outcome can provide additional insight regarding the relative contributions of habitual and goal-directed processes in guiding behavior.

The existence of dual processes appears to be advantageous in many circumstances, but one untoward consequence is that even adaptive, automatic, stimulus-elicited behaviors can be difficult to regulate. Belin et al. [3] have proposed that addiction is a pathological state arising from maladaptive habit formation, and this argument is supported by a large body of work demonstrating an association between use of addictive drugs and enhanced stimulus-response mechanisms [3]. Although there is an abundance of evidence to imply the veracity of this view, not many studies have experimentally tested whether, and under what conditions, substance-reinforced behaviors actually are insensitive to outcome devaluation (for examples, see [9], [10], [11], [12], [13], [14], [15]).

There are especially few reports, to our knowledge, regarding the effects of ethanol devaluation on ethanol-reinforced operant behaviors – and two of these [10], [14] used considerably different methods while arriving at disparate conclusions. One utilized a rat operant procedure (the ‘appetitive-consummatory’ model), which temporally segregates appetitive, instrumental actions (lever presses) from consummatory behavior (drinking a reinforcing solution) *via* use of a retractable sipper tube to grant uninterrupted access to the solution following performance of the required number of lever presses [16]. Samson et al. found that operant lever pressing conditioned by an ethanol solution according to this method was suppressed following co-administration (pairing) of oral ethanol gavage and intraperitoneal (i.p.) lithium chloride (LiCl), which causes malaise [14]. In contrast, Dickinson and colleagues [10] observed evidence that ethanol seeking was relatively insensitive to devaluation, compared to food pellet seeking (but refer to Supporting Information Text S1 for further interpretation). In their self-administration model, a dipper delivered aliquots of an ethanol solution to reinforce lever presses at variable intervals of time throughout the operant session. One plausible explanation for the discrepant conclusions between the two studies is suggested by Dickinson's earlier work that showed variable time interval (VI), as opposed to response ratio (VR), reinforcement schedules biased sucrose-reinforced lever pressing to be insensitive to sucrose devaluation [17]. Thus, the apparently conflicting findings of the former two studies [10], [14] might be explained by the very different response-reinforcer contingencies established by the two distinct ethanol self-administration procedures. In order to better elucidate the conditions under which ethanol-conditioned behaviors can become habitual, the present study explored this possibility by examining the effects of ethanol devaluation on instrumental lever pressing that was trained under either VI or VR schedules of reinforcement.

## Materials and Methods

### Ethics Statement

Experiments were conducted in accordance with the Guidelines for the Care and Use of Mammals in Neuroscience issued by the National Academies. All procedures were approved by the Institutional Animal Care and Use Committee of the University of Texas at Austin (current Animal Use Protocol #2011-00069).

### Animals

Male Long Evans rats weighing 200–225 g were allowed one week of habituation and daily handling after arriving at the Animal Resources Center of the University of Texas at Austin from Charles River Laboratories. Rats were group housed until commencement of behavioral training (after which they were individually housed) in a temperature-controlled room (72±4°F). Food and water was available *ad libitum*, except for as described under **Behavioral training and assessment**. Rats were weighed prior to any procedure, all of which occurred during the light phase of a 12 hour light/dark cycle.

In total, 180 rats were used in connection with the research reported here. Eighteen rats were used for the pilot experiments described in the Supporting Information Text S1. Of the 162 rats used for the extended and limited training experiments, 25 were excluded from final analysis of the data, because they did not complete training or their data were unreliable. For the extended training experiment, one VI and three VR rats were excluded for insufficient acquisition of 10S10E-reinforced behavior; four 10S10E VI and two 10S VI were excluded because of experimenter error. In the limited training experiment, two VI and two VR rats were excluded for low responding for 10S10E, eight were excluded for insufficient acquisition of 10S reinforced behavior, and one from each of the three groups was excluded because of experimenter error.

### Drugs and solutions

LiCl (Sigma-Aldrich) was dissolved in sterile saline (0.9% NaCl; Hospira) for injection of 125 mg LiCl/ml solution/kg body weight. Drinking solutions, 10% sucrose (w/v), 10% ethanol (v/v), or 10% sucrose (w/v):10% ethanol (v/v), were prepared using the appropriate proportions of ultra-pure sucrose (MP Biomedicals, LLC, Solon, OH), 95% ethanol (AAPER Alcohol and Chemical Co., Shelbyville, KY), and distilled water. Drinking solutions were stored at 4°C and prepared fresh approximately every three days.

### Operant chamber configuration

Instrumental training sessions were conducted in rat operant conditioning chambers (30.5 cm×24.1 cm×21 cm interior dimensions) with metal bar floors connected to lickometer circuits, housed inside sound attenuating cubicles in a dedicated behavioral testing room (chambers and cubicles from MedAssociates, Inc., Vermont, USA). The cubicles, modified by removal of the doors, were equipped with exhaust fans that provided ambient noise during all operant sessions. Med-PC IV software (MedAssociates, Inc.) controlled all chamber components. For the entire duration of all operant chamber sessions, a house light (at the top center of the left wall) was lit, and a 4.6 cm-wide retractable lever remained inserted into the chamber (6.35 cm above the grid floor, on the distal portion of the right wall). A retractable bottle assembly on the outside of the proximal panel of the right chamber wall held a bottle containing a drinking solution, with the sipper tube of the bottle positioned to be inaccessible from inside the chamber. When a lever press earned reinforcement (as determined by the programmed schedule in effect), the bottle assembly inserted, and then retracted 10 seconds later, the sipper tube through a hole into the chamber to allow the rat brief access to the drinking solution.

### Behavioral training and assessment

#### Conditioning of instrumental lever pressing

Water deprivation was initiated 22 hours prior to the first session in the operant chamber. In this habituation session only, both the lever and the sipper tube were inserted into the chamber for the entire session. Lever presses had no consequence, and the sipper tube contained 20 ml of 10S for *ad libitum* consumption. Approximately 24 hours later, rats received a conditioning session in which lever presses were reinforced according to a fixed ratio 1 schedule: each lever press yielded insertion of the sipper tube containing 10S for 10 seconds. Conditioning sessions were repeated daily until the lever press response was acquired. Sessions were usually 20 minutes in length, but were sometimes extended (to a maximum of 40 minutes) if a rat appeared to be on the verge of learning the lever press-10S reinforcer contingency. At the end of each conditioning session, rats were returned to their home cages and given free access to food and water for a minimum of two hours before water bottles were removed. Rats not acquiring lever pressing behavior after five conditioning sessions were excluded from the study.

#### Instrumental training with VI or VR reinforcement schedules

**Figure 1:**
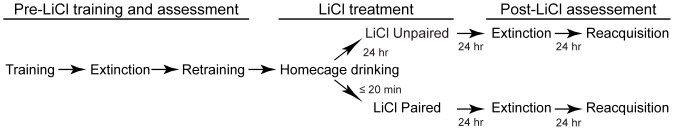
Overview of experimental phases and design. *Pre-LiCl training and assessment*: Initial training (detailed in Table 1) was followed by one (limited training) or more (extended training) cycles of extinction and retraining. Data from the last extinction and retraining sessions were used for pre-LiCl measures. *LiCl treatment*: The day after the last retraining session, all animals received the appropriate drinking solution in their home cage. LiCl injections were given either at the end of the home cage drinking period (paired treatment condition), or given 24 hours later (unpaired treatment condition). *Post-LiCl assessment*: The test for habitual behavior occurred 24 hours after LiCl injection, and was followed 24 hours later by the reacquisition test.

After acquiring the operant lever press response, rats were no longer water deprived, and began baseline instrumental training, receiving one 20 minute operant session per day, five-seven days per week (Figure 1). Table 1 shows the progression of reinforcement schedules and drinking solutions across training sessions. Slight variations to the protocol outlined in Table 1 occurred because of the *a priori-*determined requirement that the dose of ethanol be at least 0.3 g/kg for a minimum of two of the last three baseline sessions before advancement to the next phase of the experiment. For the extended training 10S10E VI and VR groups, additional sessions were conducted until reaching this criterion, and the number of sessions the 10S10E VI group received was matched by the 10S VI group. The limited training groups received a maximum of one additional session.

**Table 1:** Experimental groups and representative sequence of training parameters.

			Session Number							
Training	Group	Parameter^a^^, ^ b	1	2	3	4	5	6	7	8^c^
Extended	10S10E VI	interval	7	7	7	7	7	15	15	30
		% ethanol (v/v)	0	0	0	10	10	10	10	10
	10S VI	interval	7	7	7	7	7	15	15	30
		% ethanol (v/v)	0	0	0	0	0	0	0	0
	10S10E VR	ratio	1	1	1	1	5	5	5	10
		% ethanol (v/v)	0	0	10	10	10	10	10	10
Limited	10S10E VI	interval	7	7	7	7	15	30	30	30
		% ethanol (v/v)	0	0	10	10	10	10	10	10
	10S VI	interval	7	7	7	7	15	30	30	30
		% ethanol (v/v)	0	0	0	0	0	0	0	0
	10S10E VR	ratio	1	1	1	1	3	3	3	3
		% ethanol (v/v)	0	0	10	10	10	10	10	10

a Values listed for the reinforcement schedules are the average of the programmed values used to determine the time interval (seconds) before a single press yielded reinforcement for the VI groups, or the number of lever presses required to yield reinforcement for the VR groups. From session 7 on, the average ratio was 5 for some VR animals (n = 2 extended training, 11 limited training).

b Drinking solutions were always 10% sucrose (w/v), with either 0% or 10% ethanol (vol/vol), as shown. In the limited training 10S10E groups, some animals received one additional session with 10S before transitioning to 10S10E.

c Extended training continued with the same parameters for an additional 9 sessions, and limited training with no more than 1 additional session, before extinction and retraining (depicted in Figure 1 and described in Behavioral methods).

#### Pre-LiCl behavioral assessment and retraining

Following Samson et al. [14], extinction responding was assessed prior to LiCl treatment. On the day after the last session of the initial baseline training period, an 8 minute extinction session was conducted. During this session, a bottle containing the appropriate solution was present outside the self-administration chamber in the retracted bottle holder, but lever presses did not yield access to the sipper tube. This was followed by two (limited training) or three (extended training) sessions of retraining with the appropriate reinforcement schedule and drinking solution. Consumption of at least 0.3 g ethanol per kg body weight during at least two of the retraining sessions was required before advancement to the next phase of the experiment. Regardless of the number of baseline extinction sessions, the (final) extinction session prior to LiCl treatment was used for comparison with the extinction test conducted after LiCl treatment.

Samson and colleagues [14] suggested that repeated extinction and retraining sessions could give a better estimate of pretreatment extinction responding; thus the initial experiments utilized two cycles of extinction and retraining. The results from earlier pilot studies and the VI extended training animals showed that approximately 1/3 of animals exhibited more than ±40% change from the first to second extinction session (data not shown). Because of a concern that such variability in pretreatment extinction responding could result in reduced power to observe a devaluation effect, some animals in the VR extended training group received additional cycles of extinction and retraining until stable pressing (less than ±40% change between extinction session) was observed. However, subsequent statistical analysis of the data from all extended training groups did not indicate that stability of extinction behavior across multiple sessions was a factor influencing whether or not a devaluation effect was observed. Therefore, all limited training animals received only one cycle of extinction and retraining before LiCl treatment. Regardless of the number of baseline extinction sessions, the (final) extinction session prior to LiCl treatment was used for comparison with the extinction test conducted after LiCl treatment.

### LiCl treatment/devaluation

LiCl treatment commenced 24 hours after the final retraining session (Figure 1). Each animal was weighed and then returned to its home cage, after which the water bottle was replaced by a bottle containing the drinking solution that previously had been used as the operant reinforcer. The dose (of ethanol) and/or amount (of sucrose) that could be consumed during this procedure was limited by filling the bottle to a volume equal to the maximum the individual rat had consumed within any of the last two-three retraining sessions, plus one additional ml to compensate for loss/leakage of fluid. Animals had 20 minutes of access to the bottle, and ‘Paired’ LiCl treatment (125 mg/ml/kg, i.p. injection) was administered at the end of this period. The exception to this was the 10S limited training group, which had a maximum of 10 minutes of access before receiving paired LiCl treatment. Any rat in this group that consumed the entire volume of 10S in the bottle in less than 10 minutes was injected with LiCl immediately. For all groups, ‘Unpaired’ LiCl injections were given exactly 24 hours after the home cage consumption. Refer to the Supporting Information Text S1 for description and discussion of the pilot experiments in which the LiCl treatment procedure was optimized to elicit outcome specific devaluation by paired injections only.

### Behavioral testing

For all groups, one 8 minute extinction test (lever presses did not yield sipper tube access) was conducted exactly 24 hours after the LiCl injection (Figure 1). Twenty-four hours after the extinction test was the test for reacquisition of operant behavior. In this 20 minute operant session, animals received response-contingent reinforcement with the appropriate solution according to the schedule used prior to LiCl treatment.

### Data collection, representation, and analysis

During any session in the operant chamber, Med-PC IV software recorded the occurrence and time of event for each lever press, insertion of the sipper tube (reinforcer delivery), and lickometer circuit completion (one lick of the sipper tube). Occasionally, it was noted that the lickometer circuit appeared to not record all licks during a session (due to faulty wiring or improper placement of the sipper tube and bottle in the holder). Any animal for which this was noted was not included in lick analyses (n = 3 extended, 11 limited).

At the end of every session in the operant chamber, the total volume of remaining drinking solution (leaked solution collected by a plastic tray placed under the bottle assembly plus solution in the bottle and sipper tube) was manually measured and recorded. For the home cage presentation, the volume of any solution remaining in the tube and bottle was measured manually, but we could not recover any solution that leaked from the tube into the bedding below. Estimates of ethanol (g) consumption were calculated by subtracting the recovered volume from the initial volume of the drinking solution, and multiplying this difference by 0.0774.

Raw data from MedPC output files and paper training logs were imported, copied, or entered into Excel (Microsoft Office 2007). Excel, Prism 5 (GraphPad Software Inc.), and Adobe Illustrator CS5 (version 15.0.0) were used to create graphical representations of data (depicted as mean ± s.e.m). SPSS Statistics (versions 17.0 and 19; IBM) was used to perform general linear model procedures as appropriate. Behavioral measures from the post-LiCl tests were analyzed in two ways. Each measure was expressed as % of the pre-LiCl session for between-groups comparisons of LiCl treatment conditions (paired vs. unpaired). Additionally, raw data collected during pre- and post-LiCl sessions were used in mixed model repeated measures analyses that tested for an interaction of session (pre or post LiCl) with LiCl treatment condition (paired or unpaired).

## Results

### Adaptation of sipper tube procedure

Previous work from this lab (e.g., [18], [19], [20], [21], [22], [23], [24]) employed an appetitive-consummatory model of operant ethanol self-administration, based on the model of Samson et al. [16]. At the outset of the current study, it was unknown to us if a sipper tube method of reinforcer delivery would maintain operant responding for, or self-administration of, drinking solutions under variable reinforcement schedules. Initial pilot experiments (described in the Text S1) indicated that we could successfully adapt our existing sipper tube procedures to a new protocol, in which rats were trained to lever press for access to a sipper tube of 10% sucrose (10S) or 10% ethanol (10E) under a VI schedule. However, we observed markedly higher lever press rates for 10S than for 10E, which raised concern that comparisons of ethanol-reinforced behavior with naturally-reinforced behavior would be confounded by the large divergence in the rates of responding for 10S and 10E. Additionally, the average dose of ethanol (0.42 g/kg) administered per session was comparable to the range (approximately 0.42–0.5 g/kg) reported by other studies of 10E operant self-administration [9], [24], [25]. In those studies, the corresponding range of blood alcohol concentrations was 0.03–0.035%, suggesting that the dose of ethanol self-administered by rats drinking 10E in our self-administration model would have debatable subjective or pharmacological effects. These, and other relevant observations from our pilot experiments (refer to Text S1), guided the design of the subsequent main experiments, in which we studied behavior reinforced by a mixed sucrose and ethanol solution (10% sucrose/10% ethanol, 10S10E) relative to that reinforced by sucrose (10S) alone.

### Extended training experiment

#### Instrumental responding and self-administration

**Figure 2:**
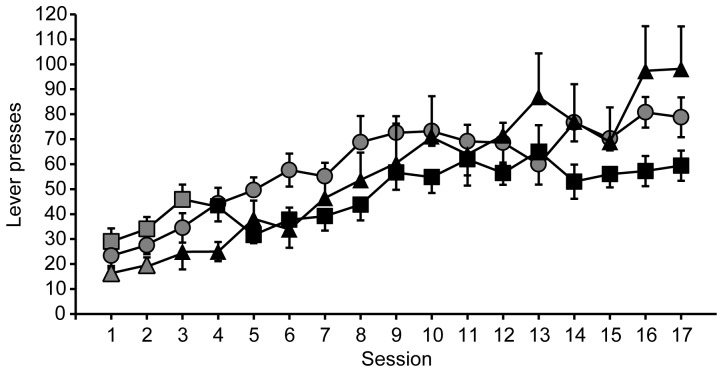
Extended instrumental lever press training. Total presses per session, for each group (10S10E VI, squares; 10S VI, circles; and 10S10E VR, triangles) across baseline training. Symbols filled with grey indicate sessions with 10S reinforcement; black filled symbols represent sessions with 10S10E reinforcement. Table 1 describes the progression of reinforcement schedules.

In the first main experiment, rats received extended instrumental training to lever press for the opportunity to self-administer 10S10E, under a VI (10S10E VI, n = 20) or a VR schedule (10S10E VR, n = 11), or 10S, under a VI schedule (10S VI, n = 19). Figure 2 shows lever pressing by each group across training sessions that correspond to the progression of schedules and drinking solutions described by Table 1. At the end of this training sequence, rats were assessed for rates of responding under extinction conditions, and then were retrained. Although some animals in the 10S10E VR group received more training sessions than the VI groups, there was no difference in body weight (P = 0.08) between the three groups at the end of training (Table 2). The total number of reinforcer deliveries (regardless of drinking solution) was similar between the 10S10E VI and 10S VI groups, but the 10S10E VR group received significantly less reinforcement across all training sessions than both VI groups (Table 2). The 10S10E VI and VR groups also were not well matched on measures of ethanol self-administration: the total number of 10S10E reinforcers received across all training sessions, and the average dose of ethanol self-administered during the final three retraining sessions were significantly less in the 10S10E VR training group (Table 2). The day after the final retraining session, rats were presented with the appropriate drinking solution in their home cages, followed by either paired (immediate) or unpaired (24 hours later) LiCl treatment. The doses consumed during home cage presentation of 10S or 10S10E are reported in Table 3.

**Table 2:** Body weight and self-administration measures prior to LiCl treatment.

Training	Group	Body weight	Total reinforcers	10S10E reinforcers	Ethanol dose
Extended	10S10E VI	516±11	396±23	334±19	1.14±0.08
	10S VI	505±13	459±19	N/A	N/A
	10S10E VR	517±18	239±25^a^	207±23^b^	0.79±0.09^c^
Limited	10S10E VI	401±6	141±8	105±6	0.91±0.06
	10S VI	399±7	164±8	N/A	N/A
	10S10E VR	416±5	148±8	100±6	0.84±0.06

Body weight (g) and ethanol dose (g/kg) are for the final retraining session, occurring 24 hours before home cage presentation of the drinking solution. ‘Total reinforcers’ indicates the sum of reinforcer deliveries across all instrumental training sessions. ‘10S10E reinforcers’ indicates the sum of reinforcer deliveries for all sessions with 10S10E reinforcement. Values are mean ± s.e.m.

a , P<0.001 vs. 10S10E VI or 10S VI.

b , P<0.01 vs. 10S10E VI.

c , P<0.05 vs. 10S10E VI.

**Table 3:** Reinforcer consumption during home cage presentation.

Training	Group	Pairing condition	Sucrose	Ethanol
Extended	10S10E VI	Paired	1.43±0.12	1.11±0.10
		Unpaired	1.18±0.22	0.91±0.17
	10S VI	Paired	1.41±0.23	N/A
		Unpaired	0.95±0.26	N/A
	10S10E VR	Paired	0.95±0.10	0.73±0.08
		Unpaired	0.96±0.25	0.74±0.19
Limited	10S10E VI	Paired	1.28±0.12	0.99±0.09
		Unpaired	1.02±0.16	0.80±0.12
	10S VI	Paired	1.52±0.11	N/A
		Unpaired	1.24±0.17	N/A
	10S10E VR	Paired	1.18±0.14	0.91±0.11
		Unpaired	1.10±0.14	0.85±0.11

Estimated consumption (in grams per kilogram of body weight) of sucrose (10S groups) or sucrose and ethanol (10S10E groups) during the home cage access period preceding LiCl treatment. Values are mean ± s.e.m.

#### No effect of LiCl treatment on lever pressing in the absence of feedback

**Figure 3:**
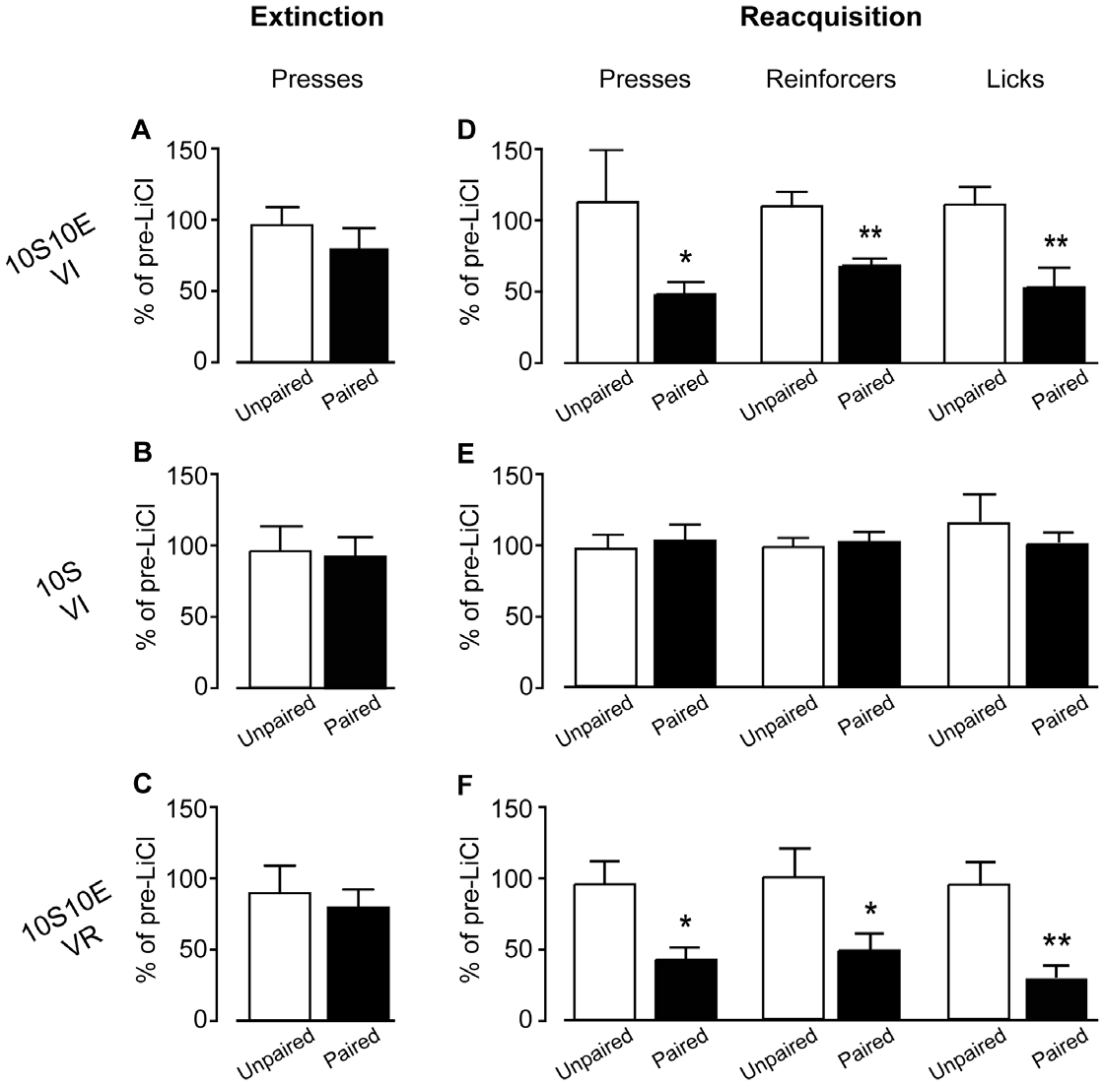
Extended training: Paired vs. unpaired LiCl treatment. (A–C): Extinction test responses (total lever presses, expressed as percent of final pre-LiCl extinction session). Paired LiCl condition (filled bars) was not significantly different from unpaired LiCl condition (open bars) in the 10S10E VI (A), the 10S VI (B), or the 10S10E VR (C) group. (D–F): Reacquisition test measures (totals, expressed as percent of final pre-LiCl training session): lever presses (left), reinforcers delivered (center) and licks (right). All measures were significantly reduced in the paired LiCl condition (filled bars) compared to the unpaired LiCl condition (open bars), for the 10S10E VI (D) and 10S10E VR (F) groups, but not for the 10S VI group (E). *, P<0.05; **, P<0.01, paired vs. unpaired. Bars represent mean+s.e.m.

**Figure 4:**
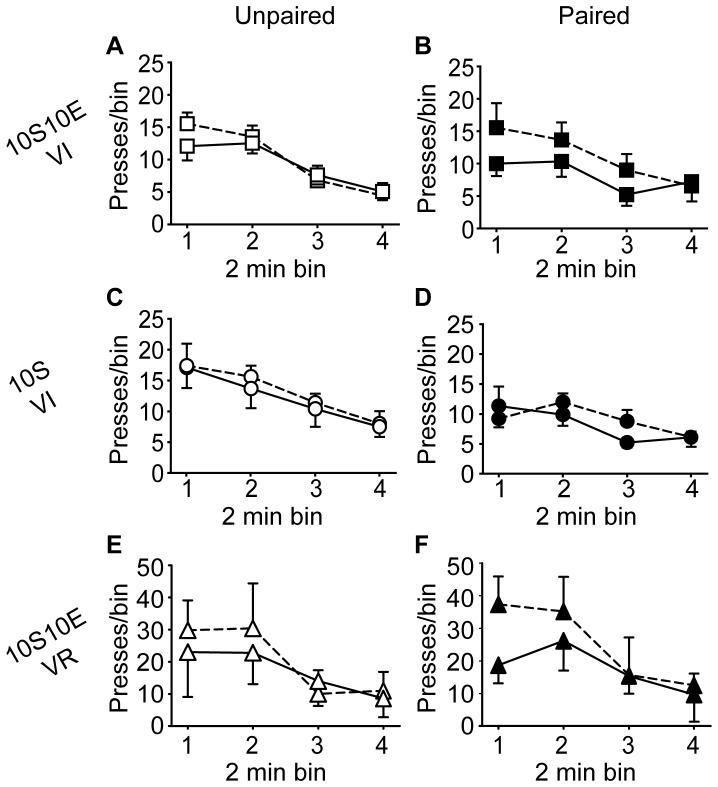
Extended training: Instrumental seeking behavior under extinction conditions. (A, C, E): Lever presses (per 2 minute bin of non-reinforced session) for the unpaired conditions (open symbols) of the 10S10E VI (A), 10S VI (C), and 10S10E VR (E) groups, before (dashed line) and after (solid line) LiCl treatment. (B, D, F): Lever presses (per 2 minute bin of non-reinforced session) for the paired conditions (filled symbols) of the 10S10E VI (B), 10S VI (D), and 10S10E VR (F), before (dashed line) and after (solid line) LiCl treatment.

To test for habitual seeking behavior after extended operant training, we evaluated the sensitivity of lever pressing to reinforcer devaluation during an extinction session in which lever presses yielded no outcome. As shown in Figure 3, extinction test lever press behavior (% of pre-LiCl extinction session) was not significantly different between the LiCl-unpaired and paired treatment conditions, for any of the three training groups, 10S10E VI (Figure 3 A, P = 0.35), 10S VI (Figure 3 B, P = 0.85), or 10S10E VR (Figure 3 C, P = 0.66). The raw data are shown in Figure 4, for each group and treatment condition, as the number of lever presses per two minute bin of the pre-LiCl session (dashed lines) and the post-LiCl test (solid lines). Mixed model analyses of these raw data were consistent with the comparisons of the normalized data; there was no interaction effect between the session (pre vs. post LiCl) and LiCl treatment condition (paired or unpaired) for any group (session×condition: 10S10E VI, P = 0.99; 10S VI, P = 0.28; 10S10E VR, P = 0.50).

#### Effects of LiCl treatment in test with response contingent feedback

Twenty-four hours after the extinction test, in which rats did not receive reinforcer feedback, all groups were tested for reacquisition of operant behavior during a session with response-contingent feedback. Figure 3 D shows that all three measures of interest were significantly reduced in the 10S10E VI animals that received paired LiCl (n = 9), relative those that received unpaired LiCl (n = 11) (presses: P = 0.03, reinforcers: P = 0.004, licks: P = 0.01). In contrast, analyses of these same measures between the paired (n = 9) and unpaired (n = 10) 10S VI group did not indicate that the LiCl-pairing affected seeking or consumption 10S (Figure 3 E; presses: P = 0.77, reinforcers: P = 0.46, licks: P = 0.54). For the 10S10E VR group, comparisons of the paired (n = 6) and unpaired (n = 5) LiCl treatment conditions were similar to those observed for the 10S10E VI group, with all measures significantly reduced in the paired condition (Figure 3 F; lever presses: P = 0.014, reinforcers: P = 0.046, licks: P = 0.004).

**Figure 5:**
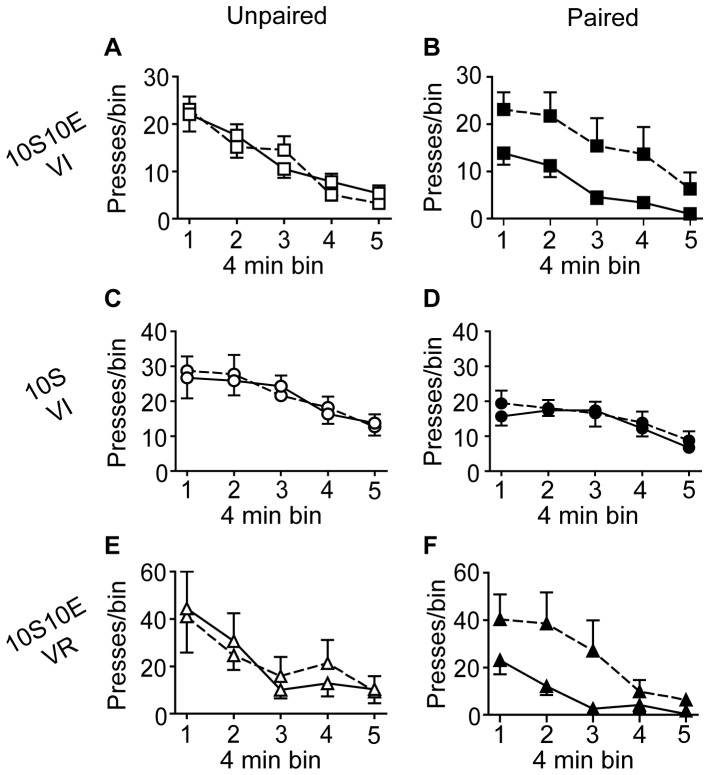
Extended training: Instrumental seeking behavior. (A, C, E): Lever presses (per 4 minute bin of reinforced session) for the unpaired conditions (open symbols) of the 10S10E VI (A), 10S VI (C), and 10S10E VR (E) groups, before (dashed line) and after (solid line) LiCl treatment. (B, D, F): Lever presses (per 4 minute bin of reinforced session) for the paired conditions (filled symbols) of the 10S10E VI (B), 10S VI (D), and 10S10E VR (F) groups before (dashed line) and after (solid line) LiCl treatment.

**Figure 6:**
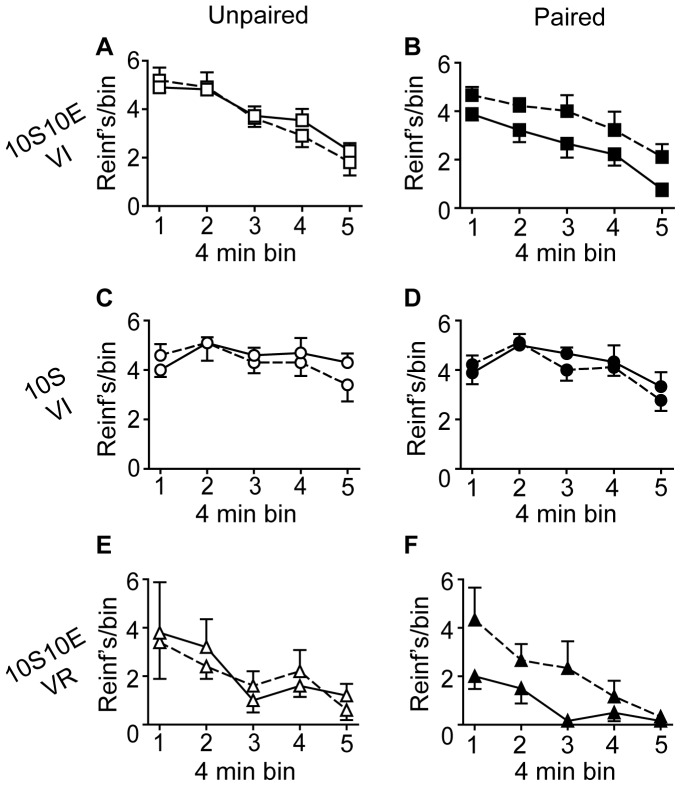
Extended training: Reinforcer deliveries. (A, C, E): Reinforcers (per 4 minute bin of reinforced session) for the unpaired conditions (open symbols) of the 10S10E VI (A), 10S VI (C), and 10S10E VR (E) groups, before (dashed line) and after (solid line) LiCl treatment. (B, D, F): Reinforcers (per 4 minute bin of reinforced session) for the paired conditions (filled symbols) of the 10S10E VI (B), 10S VI (D), and 10S10E VR (F) groups before (dashed line) and after (solid line) LiCl treatment.

**Figure 7:**
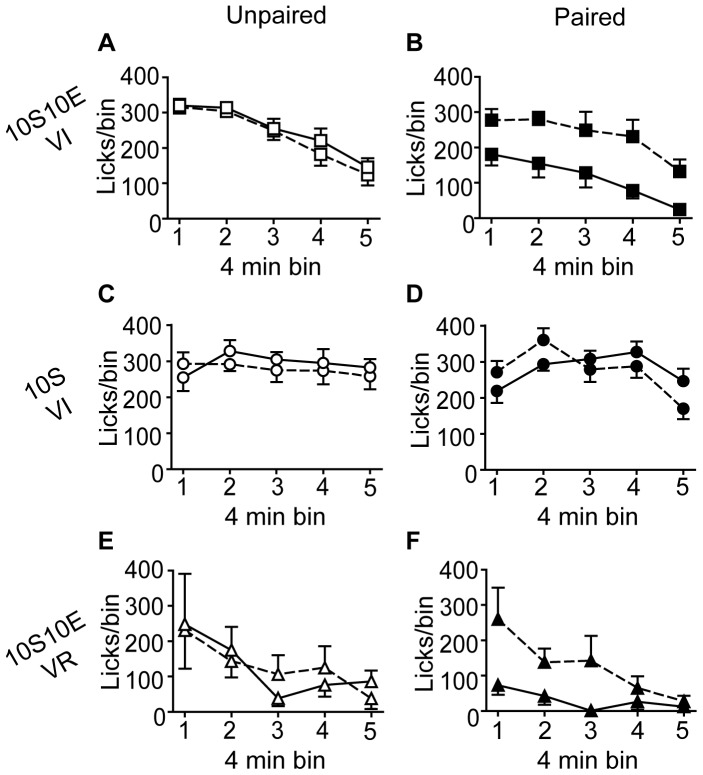
Extended training: Consummatory behavior. (A, C, E): Licks (per 4 minute bin of reinforced session) for the unpaired conditions (open symbols) of the 10S10E VI (A), 10S VI (C), and 10S10E VR (E) groups, before (dashed line) and after (solid line) LiCl treatment. (B, D, F): Licks (per 4 minute bin of reinforced session) for the paired conditions (filled symbols) of the 10S10E VI (B), 10S VI (D), and 10S10E VR (F) groups before (dashed line) and after (solid line) LiCl treatment.

The number of lever presses, reinforcer deliveries, and licks at the sipper tube per four minute bin of the pre-LiCl retraining session (dashed lines) and the post-LiCl reacquisition test (solid lines) are plotted in Figures 5, 6, and 7, respectively. Mixed model analyses of these data were consistent with the analyses of the data expressed as % of pre-LiCl. Paired LiCl treatment produced a selective devaluation of the 10S10E solution in the 10S10E VI group (session×condition: presses, P = 0.02; reinforcers, P = 0.01; licks, P = 0.01) and the 10S10E VR group (session×condition: presses, P = 0.10; reinforcers, P = 0.08; licks, P = 0.04), but LiCl treatment had no apparent effect on the value of the 10S solution in the 10S training group, regardless of pairing condition (session×condition: presses, P = 0.70; reinforcers, P = 0.43; licks, P = 0.81).

#### Discussion

For both groups that received extended training with 10S10E reinforcement, pairing of LiCl-induced malaise with consumption of 10S10E did not affect unreinforced lever pressing during the extinction test, but it did suppress 10S10E-reinforced behaviors during the reacquisition test. This pattern of results is consistent with habitual seeking behavior by both the 10S10E VI and the 10S10E VR group, which is not what we predicted. Although ratio reinforcement schedules, in general, are used to condition goal-directed behavior, the length of instrumental training can affect the sensitivity of the behavior to outcome devaluation [26]. On the other hand, these analyses were performed after training only one cohort of rats with the VR protocol. Thus, while it is possible that overtraining of the 10S10E VR group produced habitual behavior, it is also arguable that the failure to observe an effect on extinction behavior might simply be a consequence of insufficient statistical power, due to the small sample sizes. Irrespective of this matter, another fault of this experiment was that self-administration parameters were not well matched between the 10S10E groups.

With regard to the 10S group, which also showed no effect of LiCl during the test for habitual behavior, the absence of any significant effects during the reacquisition test suggests that the apparent insensitivity of unreinforced responding during the extinction test might be attributed to a failure of the LiCl pairing to devalue 10S. A parsimonious explanation for why LiCl pairing reduced 10S10E-reinforced, but not 10S-reinforced, behaviors relates to the timing of the injection. Most rats tended to drink the entire volume within 10 minutes of receiving access to the drinking solution, but LiCl was injected 20 minutes after the bottle was presented. Presumably, the salient properties of 10S were the sensory stimuli experienced during consumption the fluid; thus, we suggest that the delay between consumption of 10S and the onset of LiCl-induced malaise was too great for an aversion to be conditioned with only one pairing.

In sum, the nature of the results is such that the validity of the novel training and testing procedures used in this experiment cannot be established. Without valid measures from appropriate control groups, we are unable to determine whether comparable operant training with 10S, as opposed to 10S10E, would engender behavior insensitive to outcome devaluation, nor can we speculate regarding the relative contribution of sucrose versus ethanol to 10S10E-reinforced behaviors. Therefore, we treat the results of this experiment only as initial evidence to justify further investigation of the idea that ethanol–reinforced behaviors can become insensitive to the devaluation of their outcome. The next (limited training) experiment was designed to address the aforementioned shortcomings of this experiment.

### Limited training experiment

#### Instrumental responding and self-administration

**Figure 8:**
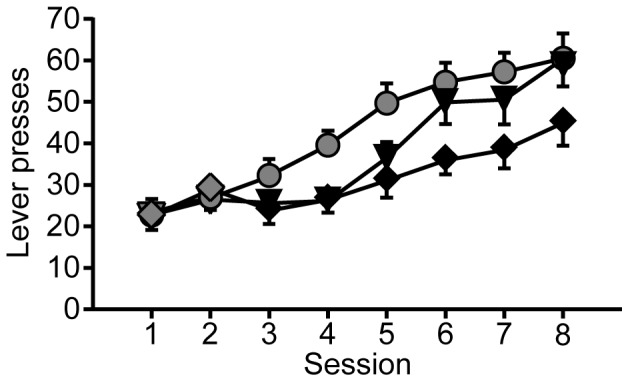
Limited instrumental lever press training. Total presses per session, for each group (10S10E VI, diamonds; 10S VI, circles; and 10S10E VR, triangles) across baseline training. Symbols filled with grey indicate sessions with 10S reinforcement; black filled symbols represent sessions with 10S10E reinforcement. Table 1 describes the progression of reinforcement schedules.

The general experimental phases and design were similar for the next experiment, in which we increased and balanced the group sizes (n = 30, 10S10E VI; 28, 10S VI; and 29, 10S10E VR), and made a few procedural modifications in light of the results of the extended training experiment. We limited the number of 10S10E-reinforced sessions to nine, and the 10S VI group received 10S for every session that the other two groups received 10S10E. We also reduced the average ratio for reinforcement of lever pressing by the 10S10E VR group (3 or 5 presses/reinforcer delivery) from that used for the extended training 10S10E VR group (10 presses/reinforcer delivery). Figure 8 shows the lever presses by each group during the training sequences described in Table 1. At the end of the limited training protocol, there were no group differences in body weights (Table 2). With the reduced VR requirement, the total number of reinforcers received across all of training was similar for all three groups. Additionally, there was no difference in the total number of 10S10E reinforcers received across training by the two 10S10E groups, who also consumed similar doses of ethanol during the final pre-LiCl session (Table 2). As a final improvement on the methodology of the extended training experiment, we modified slightly the protocol for eliciting devaluation of 10S by administering the paired LiCl injections no more than 10 minutes after introduction of 10S in the home cage. For all three groups, self-administration of the drinking solution during the home cage access period (Table 3) was comparable to the final operant session.

#### Effects of LiCl treatment on unreinforced responding

**Figure 9:**
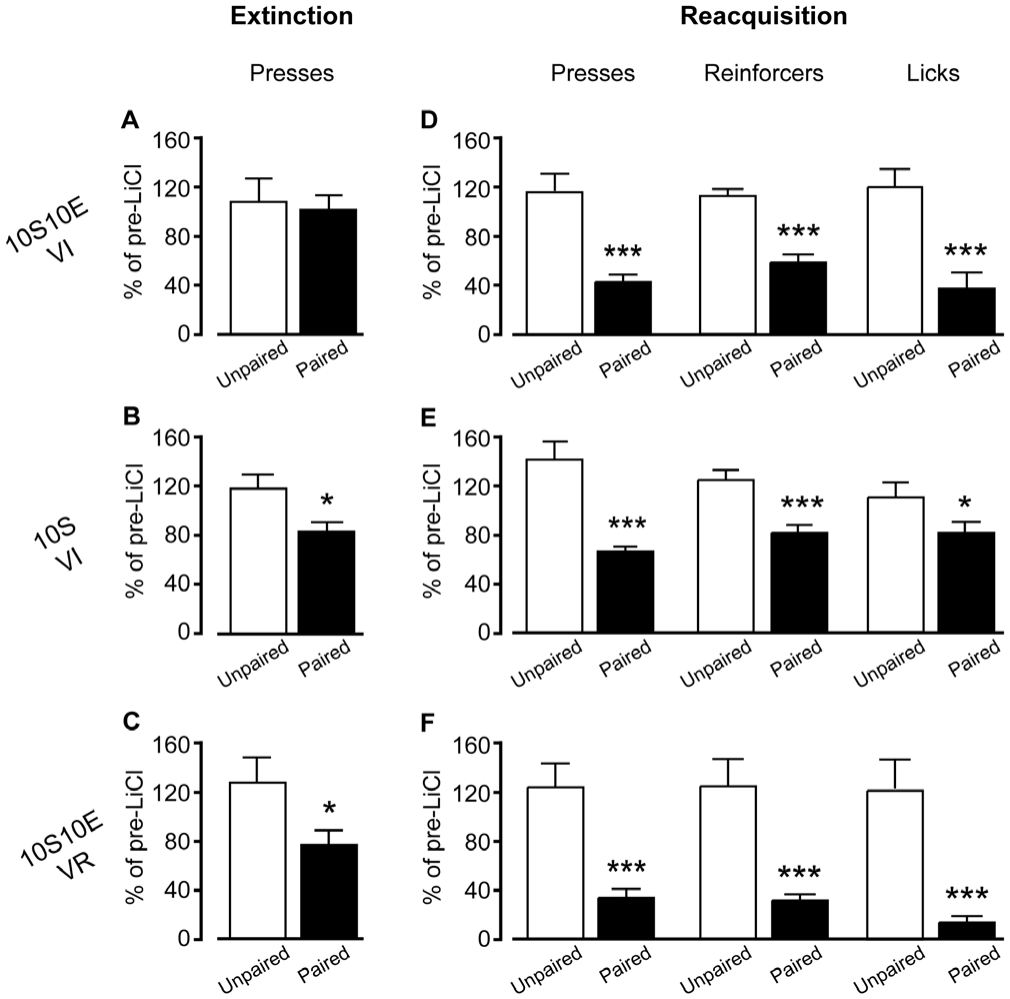
Limited training: Paired vs. unpaired LiCl treatment. (A–C): Extinction test responses (total lever presses, expressed as percent of pre-LiCl session). Paired LiCl condition (filled bars) was not different from unpaired LiCl condition (open bars), for the 10S10E VI group (A), but was for the 10S VI (B) and the 10S10E VR (C) groups. (D–F): Reacquisition test measures (totals, expressed as percent of final pre-LiCl training session): lever presses (left), reinforcers delivered (center) and licks (right). All measures were significantly reduced in the paired LiCl condition (filled bars) compared to the unpaired LiCl condition (open bars), for the 10S10E VI (D), the 10S VI (E), and the 10S10E VR (F) groups. *, P<0.05; ***, P<0.001, paired vs. unpaired. Bars represent mean+s.e.m.

**Figure 10:**
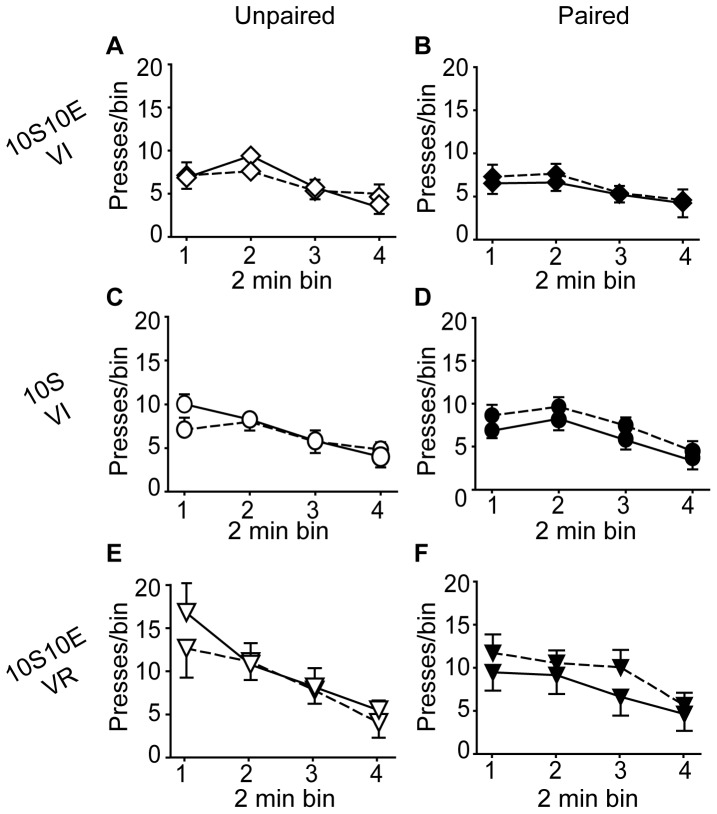
Limited training: Instrumental seeking behavior under extinction conditions. (A, C, E): Lever presses (per 2 minute bin of non-reinforced session) for the unpaired conditions (open symbols) of the 10S10E VI (A), 10S VI (C), and 10S10E VR (E) groups, before (dashed line) and after (solid line) LiCl treatment. (B, D, F): Lever presses (per 2 minute bin of non-reinforced session) for the paired conditions (filled symbols) of the 10S10E VI (B), 10S VI (D), and 10S10E VR (F), before (dashed line) and after (solid line) LiCl treatment.

During the test for sensitivity to outcome devaluation (extinction test), there was no difference in lever pressing (expressed as % of pre LiCl in Figure 9 A–C) between the paired (n = 17) and unpaired (n = 13) LiCl conditions of the 10S10E VI group (P = 0.53). In contrast, lever pressing was significantly reduced in the paired conditions of the 10S VI (n = 17) and 10S10E VR (n = 16) groups, relative to the unpaired conditions (n = 11, 10S VI; 13, 10S10E VR) of each group (P = 0.026, 10S VI; 0.039, 10S10E VR). To rigorously evaluate if the type of reinforcer or the reinforcement schedule were statistically significant factors affecting the sensitivity of lever pressing to outcome devaluation, we performed 3-way mixed model analyses of the raw data (plotted in Figure 10 as presses per 2 minute bin). When comparing the two VI trained groups, the interaction of session (pre vs. post LiCl)×condition (paired vs. unpaired)×reinforcer (10S10E vs. 10S) was not significant (P = 0.25). Similarly, analysis of session×condition×schedule (VI vs. VR), for the 10S10E reinforced groups, failed to reach significance (P = 0.16). Nevertheless, we did find support for our *a priori* hypothesis that, within each group, 2-way analysis the session and condition factors would show a significant interaction between these terms for the 10S VI (P = 0.039) and the 10S10E VR (P = 0.039) groups, but not for the VI 10S10E group (P = 0.42).

#### Effects of LiCl treatment in test with response contingent feedback

**Figure 11:**
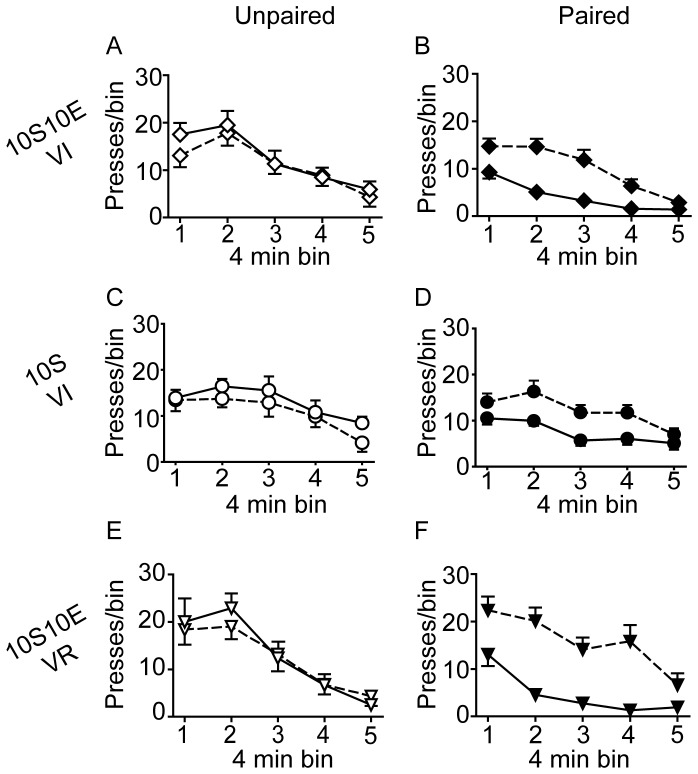
Limited training: Instrumental seeking behavior. (A, C, E): Lever presses (per 4 minute bin of reinforced session) for the unpaired conditions (open symbols) of the 10S10E VI (A), 10S VI (C), and 10S10E VR (E) groups, before (dashed line) and after (solid line) LiCl treatment. (B, D, F): Lever presses (per 4 minute bin of reinforced session) for the paired conditions (filled symbols) of the 10S10E VI (B), 10S VI (D), and 10S10E VR (F) groups before (dashed line) and after (solid line) LiCl treatment.

**Figure 12:**
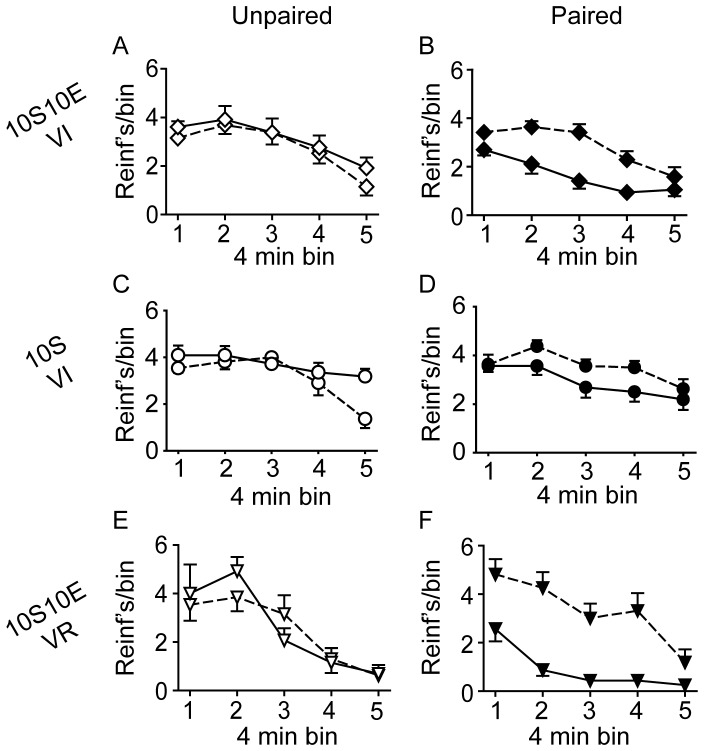
Limited training: Reinforcer deliveries. (A, C, E): Reinforcers (per 4 minute bin of reinforced session) for the unpaired conditions (open symbols) of the 10S10E VI (A), 10S VI (C), and 10S10E VR (E) groups, before (dashed line) and after (solid line) LiCl treatment. (B, D, F): Reinforcers (per 4 minute bin of reinforced session) for the paired conditions (filled symbols) of the 10S10E VI (B), 10S VI (D), and 10S10E VR (F) groups before (dashed line) and after (solid line) LiCl treatment.

**Figure 13:**
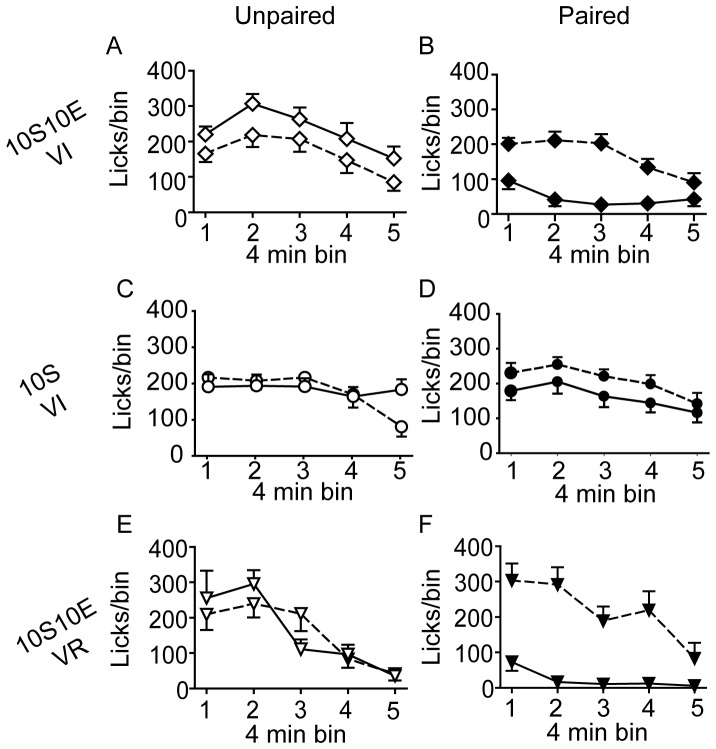
Limited training: Consummatory behavior. (A, C, E): Licks (per 4 minute bin of reinforced session) for the unpaired conditions (open symbols) of the 10S10E VI (A), 10S VI (C), and 10S10E VR (E) groups, before (dashed line) and after (solid line) LiCl treatment. (B, D, F): Licks (per 4 minute bin of reinforced session) for the paired conditions (filled symbols) of the 10S10E VI (B), 10S VI (D), and 10S10E VR (F) groups before (dashed line) and after (solid line) LiCl treatment.

When given response contingent feedback during the reacquisition test, all groups showed clear evidence of behavioral suppression as a result of LiCl pairing. Significant differences (all P<0.001) between the paired and unpaired LiCl treatment conditions, for all three behavioral measures of interest (expressed as % of pre LiCl), were observed for both the 10S10E VI (Figure 9 D) and the 10S10E VR (Figure 9 F) groups. Lever presses (P<0.001), reinforcer deliveries (P = 0.001), and licks (P = 0.047) also were reduced in the paired condition of the 10S VI group, relative to unpaired (Figure 9 E). Mixed model analyses of the raw data (plotted in 4 minute bins in Figures 11–13) confirmed that LiCl treatment suppressed behavior from the pre-LiCl session (dashed lines) to the post-LiCl test (solid lines) in the paired conditions (filled symbols), but not the unpaired condition (open symbols). For all three groups, the interaction effect of session×treatment condition was at the P≤0.001 level of significance for lever presses (Figure 11), reinforcers (Figure 12), and licks (Figure 13). The exception to this was licking behavior by the 10S VI group, which did not achieve significance (P = 0.109).

#### Discussion

By increasing the number of animals per group, limiting the number of training sessions for all groups, and reducing the average ratio of presses/reinforcer delivery for the 10S10E VR group, we established instrumental lever pressing in three different training groups that were matched for group size and the total number of reinforcers received in the operant context across training sessions. Despite being well matched on a number of training parameters, all three groups did not exhibit sensitivity to paired LiCl treatment when lever pressing did not receive reinforcement. In the absence of any feedback from the instrumental outcome, only lever pressing by animals in the paired LiCl treatment conditions of the 10S VI and 10S10E VR groups was significantly reduced.

The results of the reacquisition test validate the LiCl treatment procedures used for this experiment. For the unpaired conditions of each group, there were no changes in behavior between the pre LiCl session and the post LiCl test, showing that the single unpaired LiCl injection did not cause nonspecific behavioral suppression. This represents an improvement over previous studies on the effects of ethanol devaluation [10] that were confounded by nonspecific behavioral suppression or context aversion after LiCl treatment. For the paired condition of the 10S group, LiCl was administered as rats finished drinking 10S, after no more than 10 minutes of home cage access. The effectiveness of this modified pairing procedure to devalue 10S was confirmed by the decreased seeking and consumption of 10S (relative to unpaired treatment) during the reacquisition test. The rats in the 10S10E groups also consumed the entire volume of their drinking solution within approximately the same amount of time as the rats drinking 10S, and LiCl injected 20 minutes after introduction of 10S10E in the home cage suppressed seeking and consumption of 10S10E by both the VI and the VR group during the reacquisition test.

These findings suggest that devaluation of 10S by the 10 minute LiCl pairing was mediated by sensory stimuli accompanying its consumption, whereas postingestive, interoceptive stimuli mediated devaluation of 10S10E by the 20 minute LiCl pairing. Thus, we infer that the coincident LiCl-induced malaise devalued the subjective state induced by the pharmacological actions of ethanol. We do not know for certain however, if the 20 minute LiCl pairing selectively devalued only the pharmacological effects of the ethanol in the 10S10E solution. Similarly, it is questionable as to whether the 20 minute pairing of LiCl with 10S10E produced a cross-devaluation of sucrose.

Instrumental responding evaluated in the absence of response-contingent reinforcer feedback (i.e., extinction conditions) is not driven solely by instrumental learning about the most recent type of reinforcer, but rather, is influenced by all reinforcement learning (Pavlovian and instrumental) that has occurred in the test context (cf. [8], [27], [28], [29], [30], [31]). Therefore, lever pressing by both of the 10S10E groups during the extinction test likely was governed not just by 10S10E-reinforced learning, but also by 10S-reinforced learning. Nevertheless, despite receiving equivalent numbers of 10S and 10S10E reinforcers across the same number of training sessions, only the VR trained group showed a suppression of lever pressing in the extinction test after LiCl pairing with 10S10E. Thus, we argue that regardless of any contribution made by sucrose-reinforced learning to lever press performance by the 10S10E groups, the contribution made by ethanol-reinforced learning was not equally sensitive to a change in the value of ethanol.

In summary, our interpretation is that after limited training, habitual control of behavior was evident in the 10S10E VI group alone, and this was apparent only under conditions in which lever presses did not receive feedback from the devalued outcome. In other words, we infer that the neural mechanisms that enable actions to be automatically elicited, in response to stimuli other than the instrumental reinforcer itself, were able to stimulate lever pressing by the 10S10E group. However, when lever presses received contingent feedback from 10S10E, the instrumental actions of this group were modulated in accordance with the diminished value of their outcome (i.e., actions came under goal-directed control). Finally, the immediate reductions in lever press performance by the 10S VI and 10S10E VR groups after outcome devaluation implies that, in these groups, stimulation of lever press behavior was still reliant on the instrumental outcome (or its neural representation).

## General Discussion

The present report serves to confirm and extend several studies regarding the effects of outcome devaluation on instrumental responding for food, sucrose, or ethanol reinforcement. It was previously observed that lever pressing reinforced by magazine deliveries of sucrose pellets [26], food pellets [32], or 10% ethanol aliquots [9] displayed decreasing sensitivity to outcome devaluation with increasing lengths of training. The procedural differences between the extended and limited training experiments preclude us from directly analyzing the effect of training length on the sensitivity of lever pressing to outcome devaluation, but our findings are not inconsistent with this general established relationship between amount of training and sensitivity to outcome devaluation. However, it has also been noted with respect to sucrose- and food-reinforced behaviors that the operant reinforcement contingency appears to be an important factor influencing the time course by which insensitivity emerges [17], [26], [32]. In the introduction to this paper, we proposed that the apparently conflicting conclusions of Dickinson et al. [10] and Samson et al. [14], might be explained by the very different operant reinforcement contingencies established by their models of ethanol self-administration. The differential sensitivity of the 10S10E VI and VR groups of the limited training experiment lend credence to this idea.

Interestingly, however, the instrumental contingency alone cannot account for the insensitivity of the limited training 10S10E VI group, as seeking behavior by the 10S VI group was sensitive to outcome devaluation. Nevertheless, the sensitivity of lever pressing by rats that received limited VI training with 10S reinforcement does seem to indicate that the insensitivity of lever pressing to outcome devaluation after identical training with 10S10E is at least partially attributable to the addition of ethanol to the drinking solution. Thus, although the sensitivity of the 10S10E VR group refutes any explanation of our findings based solely on the effects of ethanol, we consider for a moment how ethanol exposure, in general, could influence the observable sensitivity of instrumental responding to outcome devaluation.

One possibility is that ethanol-associated contextual cues alter the expression of behavior, independently of any influence on the acquisition of instrumental behavior. For example, Ostlund et al. [33] found that food or sucrose pellet seeking was insensitive to devaluation when tested in an ethanol-associated context, but not when the same rats were tested in a saline-associated context. It is unlikely though, in respect to our study, that testing in an ethanol-associated context, *per se*, impaired the expression of goal-directed behavior after limited training, as only one of the ethanol-exposed groups was insensitive to outcome devaluation.

Alternatively, ethanol administration could alter the balance of learning by each pathway (enhance habit acquisition) during instrumental training. Corbit et al. [9] recently reported that after eight weeks of instrumental training, sucrose seeking by ethanol-naïve rats remained sensitive to outcome devaluation, but sucrose seeking by ethanol-experienced rats had become insensitive. The ethanol-experienced rats were pre-exposed to ethanol for four weeks before training, and, once training began, drank ethanol in their home cages several hours after each instrumental training session. Thus, Corbit and colleagues findings suggest that chronic exposure to low to moderate doses of ethanol (∼0.4 g/kg/session) induces changes in neural circuitry or synaptic physiology, which alone are sufficient to produce a general facilitation of habit formation. The design of our experiments does not allow us to distinguish between the contributions made by ethanol-induced neural adaptations and the acute pharmacological actions of ethanol. However, given that our rats were not pre-exposed to ethanol, were exposed for much less time (maximum of four weeks in the extended training experiment), and drank moderate to high doses of ethanol (0.7–1.2 g/kg) with sucrose in the operant chamber, it is arguable that the acute actions of ethanol experienced during instrumental training sessions played an important role in the development of habitual ethanol seeking.

In either case, we speculate that the ability of ethanol exposure to alter dopamine neurotransmission during instrumental training sessions contributed to the insensitivity of lever pressing to outcome devaluation after limited training with 10S10E reinforcement under a variable interval schedule. An intriguing possibility, suggested by our finding, is that the actions of ethanol interacted with, or enhanced, the neural mechanism(s) by which variable reinforcement schedules bias toward earlier emergence of habitual behavior. It is not completely understood how reinforcement schedules impact the balance of learning by goal-directed or habitual pathways, but work by DeRusso and colleagues indicates that variability in the temporal contiguity between instrumental actions and reinforcer delivery accounts for their influence [32]. It has been proposed that reinforcer (or reward)-evoked phasic dopamine release is able to precisely modulate spike-timing-dependent plasticity at synapses [34], [35], and that this phenomenon underlies the development of neurophysiological or behavioral responses to reward-predictive stimuli [36], [37]. Perhaps, by manipulating the timing of reinforcer receipt, variable reinforcement schedules alter the timing of the reinforcer-evoked phasic dopamine response in a way that enhances learning about reward-predictive stimuli.

Similarly, ethanol exposure or self-administration could change the precision with which dopamine modulates learning and influences behavioral responses. Acute intravenous administration of ethanol pharmacologically increases phasic dopamine release in the nucleus accumbens of rats [38], and operant self-administration of 10S10E has been associated with increased extracellular dopamine concentrations in the core-shell border of the nucleus accumbens [22], [23]. In both studies, the dopaminergic response to drinking 10S10E was absent in animals drinking 10S, and Carrillo et al. showed that this response developed after the first session with 10S10E reinforcement [23]. These studies substantiate our hypothesis that the acute responses to self-administration of 10S10E impacted learning by the animals in our experiments by enhancing dopamine neurotransmission. Moreover, this effect on dopamine release can be attributed primarily to the ethanol and not the sucrose in the 10S10E solution, and potentially accounts for the differences in behavior between the 10S10E and 10S VI groups. Finally, chronic ethanol exposure may not be necessary for the facilitation of habit formation, as ethanol-induced neural adaptations in dopamine signaling can occur after a single exposure to 10S10E in the operant context.

In short, we propose that both variable interval reinforcement schedules and ethanol exposure may enhance the ability of sensory stimuli representing reward availability to drive instrumental actions by interfering with the spatiotemporal precision of dopaminergic modulation of synaptic plasticity. This does not exclude other ways in which ethanol could alter the balance between habitual and goal-directed control of behavior, but suggests a general explanation for our finding that, after limited training, only the group that received ethanol reinforcement under a variable interval schedule exhibited lever pressing that was relatively insensitive to outcome devaluation. Future studies will have to evaluate the specific pharmacological and physiological mechanisms by which the type of reinforcer, schedule of reinforcement, and duration of training influence the emergence of habitual behavior.

## Supporting Information

Text S1 Results and discussion of pilot experiments and methods for inducing outcome devaluation by LiCl pairing.
(DOCX)
