# Supplementary material for: Ethanol Seeking by Long Evans Rats Is Not Always a Goal-Directed Behavior
Source: PLoS One. 2012 Aug 3;7(8):e42886. doi: 10.1371/journal.pone.0042886 (PMC3411727; doi:10.1371/journal.pone.0042886)
Supplement: Text S1 — Results and discussion of pilot experiments and methods for inducing outcome devaluation by LiCl pairing. (DOCX) [file pone.0042886.s001.docx]

**RESULTS AND DISCUSSION OF PILOT EXPERIMENTS**

***Pilot experiments: instrumental training methods***

We first conducted small pilot studies to evaluate if 10% ethanol (10E) or 10% sucrose (10S) in a sipper tube, delivered according to a VI schedule, would maintain operant responding and self-administration. In all cohorts, lever press behavior was conditioned by reinforcement of each press with 10 seconds of access to a sipper tube containing 10S. After acquisition of lever press behavior, the reinforcer was switched to 10S10E for the ethanol reinforced cohorts, or remained 10S for the sucrose cohorts. The concentration of sucrose in the 10S10E was faded to 10E over several sessions while the reinforcement schedule remained at VI7, after which the schedule increased to VI15 and then VI30. We found that 10 of 11 animals continued to lever press for 10E on the VI30 reinforcement schedule, with an average rate of one press/minute. In animals maintained on 10S, six out of seven rats continued to lever press as the reinforcement schedule increased from VI7 through VI15 to VI30. The average rates of responding for two different 10S cohorts were markedly higher than that of the 10E cohort – approximately five presses/minute by animals that received 14 sessions with 10S reinforcement (n = 3), or 17 presses/minute by animals that received equivalent training sessions to match the 10E animals (n = 3).

***Pilot experiments: devaluation procedure***

One commonly used method to experimentally devalue a reinforcing substance is to pair ingestion of the substance with an injection of lithium chloride (LiCl), which produces malaise. Both the Dickinson et al. [1] and Samson et al. [2] studies found that repeated reinforcer–LiCl pairings were very effective to induce an apparent change in reinforcer value. However, they also both observed LiCl-induced behavioral changes that were not solely attributable to outcome specific devaluation by LiCl treatment. For example, Dickinson and colleague’s claim regarding the relative insensitivity of ethanol-reinforced lever pressing to ethanol devaluation was not an absolute declaration, but rather, was made in relation to sensitivity to food pellet devaluation. In fact, both devaluation procedures, which paired LiCl injections with food pellet or ethanol reinforcers delivered in the operant chamber, reduced ethanol-seeking relative to the unpaired LiCl treatment procedure, which injected LiCl in the home cage unpaired with any reinforcer. Dickinson et al. argued that the more relevant comparison was between the food pellet and ethanol devalued groups than between either of these groups and the unpaired controls, because both paired groups presumably developed a general context aversion to the operant chamber, whereas the unpaired group did not. Thus, because ethanol devaluation did not have a greater effect than food pellet devaluation to reduce ethanol seeking, but food pellet devaluation did have a greater effect than ethanol devaluation to decrease food pellet seeking, the authors claimed ethanol seeking was insensitive to devaluation under conditions in which food pellet seeking was sensitive to devaluation. Nevertheless, the context aversion and outcome devaluation effects may have interacted in ways that are not readily apparent, and the generalizablity of the claim that ethanol seeking is insensitive to ethanol devaluation is unclear.

From the outset, our goal was to find unequivocal evidence to indicate if, and when, ethanol-reinforced behaviors are under the control of habitual processes. Sound conclusions regarding the sensitivity of ethanol-reinforced behavior to ethanol devaluation are not possible when the intended ‘outcome devaluation’ procedure does not selectively affect the value of the outcome. Thus, a predetermined requisite condition for our experiments was that the outcome devaluation procedure produced outcome specific devaluation, and that the apparent reduction in value was not secondary to other nonspecific LiCl-induced effects. Based on Dickinson et al.’s finding that LiCl administered after reinforcers were delivered in the operant chamber produced a general context-dependent suppression of behavior[1], we decided upon home cage access to the sipper tube of a bottle containing the appropriate drinking solution as the method for reinforcer delivery in the devaluation procedure. For the first devaluation pilot, we performed 4 cycles of treatment, during each of which a LiCl injection (125 mg/kg, i.p.) was administered either after 20 minutes of access to 10E in the home cage (paired condition, n = 2), or 24 hours post 10E home cage consumption (n = 2). With this treatment protocol, we observed evidence of non-specific behavioral suppression; operant behaviors under extinction and during multiple reacquisition sessions were decreased following 4 treatments with LiCl, regardless of condition (data not shown). This result replicated Samson and colleagues’ finding that 3 or 4 unpaired LiCl injections reduced extinction responding for 10E. In their study, LiCl injections were paired or unpaired with oral gavage of ethanol in the vivarium to avoid conditioning an operant context aversion, so Samson et al. reasoned that repeated exposure to LiCl-induced illness caused nonspecific behavioral suppression. Indeed, in addition to reduced operant performance, we also observed several signs of sickness (e.g., unkempt fur, slowed body weight gain or weight loss, and reduced exploratory activity during handling) in our cohort of animals receiving 4 injections of LiCl, so we next assessed the ability of a single LiCl pairing to devalue 10E in other cohorts of pilot animals. We found that a single pairing of LiCl with home cage 10E consumption (n = 2) reduced consumption of 10E in subsequent operant sessions, but a single unpaired LiCl injection (n=3) did not alter subsequent 10E consumption (data not shown). Based on this evidence that a single pairing with LiCl would devalue a 10E solution without producing non-specific, sickness-related effects, we used single injections of LiCl for our subsequent experiments using 10S10E and 10S solutions as operant reinforcers.

***References***

1. Dickinson A, Wood N, Smith JW (2002) Alcohol seeking by rats: action or habit? Q J Exp Psychol B 55: 331-348.

2. Samson HH, Cunningham CL, Czachowski CL, Chappell A, Legg B, et al. (2004) Devaluation of ethanol reinforcement. Alcohol 32: 203-212.
